# Molecular Insights into Rice Immunity: Unveiling Mechanisms and Innovative Approaches to Combat Major Pathogens

**DOI:** 10.3390/plants14111694

**Published:** 2025-06-01

**Authors:** Muhammad Usama Younas, Bisma Rao, Muhammad Qasim, Irshad Ahmad, Guangda Wang, Quanyi Sun, Xiongyi Xuan, Rashid Iqbal, Zhiming Feng, Shimin Zuo, Maximilian Lackner

**Affiliations:** 1 Key Laboratory of Plant Functional Genomics of the Ministry of Education, Agricultural College of Yangzhou University, Yangzhou 225009, China; usamaghias7@gmail.com (M.U.Y.); dx120210125@stu.yzu.edu.cn (G.W.); sunquanyi2023@163.com (Q.S.); opoetxiong@gmail.com (X.X.); 2 Jiangsu Key Laboratory of Crop Genomics and Molecular Breeding, Agricultural College of Yangzhou University, Yangzhou 225009, China; 3 Department of Public Health, Medical College, Yangzhou University, Yangzhou 225009, China; raobisma83@gmail.com; 4 Microelement Research Center, College of Resources and Environment, Huazhong Agricultural University, Wuhan 430070, China; qrajput64@gmail.com; 5 Joint International Research Laboratory of Agriculture and Agri-Product Safety, Ministry of Education of China, Yangzhou University, Yangzhou 225009, China; irshadgadoon737@yahoo.com; 6 Department of Agronomy, Faculty of Agriculture and Environment, The Islamia University of Bahawalpur, Bahawalpur City 63100, Pakistan; rashid.iqbal@iub.edu.pk; 7 Department of Life Sciences, Western Caspian University, Baku AZ1001, Azerbaijan; 8 Department of Industrial Engineering, University of Applied Sciences Technikum Wien, Hoechstaedtplatz 6, 1200 Vienna, Austria

**Keywords:** rice diseases, *R* genes, QTLs, CRISPR/Cas9, sustainable agriculture

## Abstract

Rice (*Oryza sativa*) is a globally important crop that plays a central role in maintaining food security. This scientific review examines the critical role of genetic disease resistance in protecting rice yields, dissecting at the molecular level how rice plants detect and respond to pathogen attacks while evaluating modern approaches to developing improved resistant varieties. The analysis covers single-gene-mediated and multi-gene resistance systems, detailing how on one hand specific resistance proteins, defense signaling components, and clustered loci work together to provide comprehensive protection against a wide range of pathogens and yet their production is severely impacted by pathogens such as *Xanthomonas oryzae* (bacterial blight) and *Magnaporthe oryzae* (rice blast). The discussion extends to breakthrough breeding technologies currently revolutionizing rice improvement programs, including DNA marker-assisted selection for accelerating traditional breeding, gene conversion methods for introducing new resistance traits, and precision genome editing tools such as CRISPR/Cas9 for enabling targeted genetic modifications. By integrating advances in molecular biology and genomics, these approaches offer sustainable solutions to safeguard rice yields against evolving pathogens.

## 1. Introduction

Rice (*Oryza sativa*) is the primary food crop source for more than half of humanity and is particularly important in Asia, where approximately 92% of the world’s rice-growing area is cultivated [1,2]. As global population projections exceed 8 billion by 2025, agricultural systems face the dual challenge of increasing yields by 50% while transitioning to more sustainable practices [3]. This imperative makes controlling disease-related yield losses not just an agronomic concern, but a critical component of global food security and sustainable development goals. Rice crops continue to face threats from various destructive pathogens that have the potential to have severe economic impacts on agricultural communities around the world [4,5]. The most destructive rice diseases include the fungal rice blast, bacterial leaf blight, sheath blight, and bacterial panicle blight [5,6], each of which presents unique production challenges (Table 1). Rice blast is particularly destructive, with documented yield losses of up to 100% under epidemic conditions; these losses disproportionately affect resource-poor regions where rice constitutes up to 70% of daily caloric intake [7]. In developing countries where rice provides 50–80% of daily calories for over 3 billion people, disease outbreaks exacerbate food insecurity by reducing harvests and increasing market prices, pushing vulnerable populations toward malnutrition. Bacterial leaf blight impairs photosynthesis through leaf damage [8], while sheath blight, which thrives in warm, humid conditions, can reduce yields by 50% [8]. Bacterial panicle blight directly compromises grain development [9]. The insect-borne Tongro virus causes stunted growth and leaf discoloration in rice, significantly reducing yield [8]. Table 1 provides a comprehensive list of the characteristics and economic consequences of these pathogens, highlighting the need for improved control measurements.

**Figure 1:**
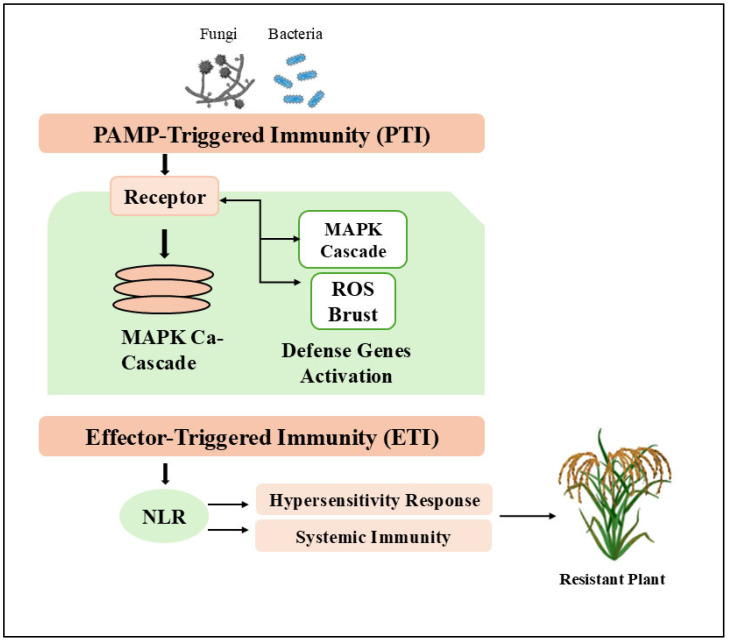
Mechanisms of plant immune responses to fungal, bacterial, and viral pathogens, including PAMP-triggered immunity (PTI), effector-triggered immunity (ETI), activation of defense genes, and systemic immunity, leading to resistance. Rice plants recognize pathogen-associated molecular patterns (PAMPs) via cell surface receptors, initiating PTI through the mitogen-activated protein kinase (MAPK) cascade and reactive oxygen species (ROS) burst, leading to defense gene activation [20,21]. Additionally, intracellular nucleotide-binding leucine-rich repeat receptor (NLR) proteins recognize specific pathogen effectors, triggering ETI characterized by hypersensitive response and systemic immunity [18,20]. Both pathways contribute synergistically to establishing resistance in rice, as represented by the outcome of a resistant plant.

Maintaining rice productivity requires an integrated disease management strategy, in which early detection is critical for timely intervention [13]. Traditional diagnostic methods rely on visual symptoms and laboratory testing, which are time-consuming and require technical expertise and therefore have limitations [14]. Understanding rice’s innate defense mechanisms provides a foundation for developing sustainable solutions. The plant immune system operates through a complex two-tiered defense strategy. The first layer, called PAMP-triggered immunity [15], is activated when cell surface receptors recognize conserved microbial patterns, triggering downstream defenses including kinase cascades, oxidative burst, and defense gene activation [16,17] (Figure 1). Pathogens counteract this by inhibiting the effector proteins of PTI, thereby establishing effector-triggered susceptibility [18]. Rice plants overcome this disruption by detecting resistance proteins of pathogen effectors, initiating a more robust effector-triggered immunity response characterized by local cell death and systemic resistance [18]. Modern breeding programs exploit these natural defense systems to develop resistant varieties to reduce reliance on pesticides while ensuring stable production [19].

**Figure 2:**
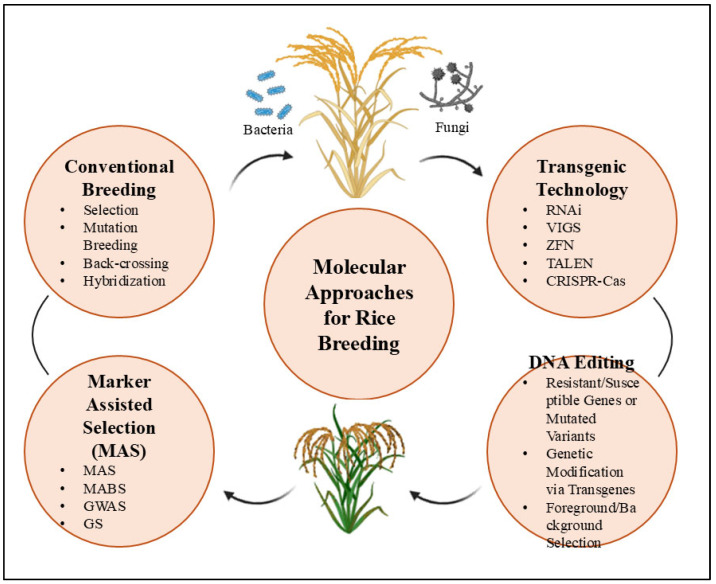
The figure outlines four key approaches: (1) conventional breeding (selection, mutation, hybridization, and back-crossing) [29]; (2) marker-assisted selection (MAS, including marker-assisted backcrossing (MABS), genome-wide association studies (GWAS), and genomic selection (GS)) [30]; (3) transgenic technologies (RNA interference (RNAi) and virus-induced gene silencing (VIGS)) [31]; and (4) DNA editing (identification of resistant/susceptible genes or mutated variants, genetic modification via transgenes, and foreground/background selection) [32]. These methods collectively target resistance mechanisms against bacterial, viral, and fungal pathogens in rice.

Modern breeding programs exploit these natural defense mechanisms to develop varieties that reduce pesticide use by 30–50% while maintaining or improving yields [22,23]. Contemporary breeding combines three complementary approaches: conventional hybridization techniques [24], DNA marker-assisted selection, and genetic engineering methods [25,26] (Figure 2). Key to these efforts is the identification and characterization of resistance genes through molecular mapping, gene cloning, and transgenic line development [27]. While natural genetic variation provides the necessary resources, breeders complement this through mutagenesis and targeted genetic modification to overcome the limitations of available diversity [28].

This review systematically examines genetic resistance mechanisms in rice, focusing on molecular interactions between the host and pathogen, genetic determinants of immunity, signaling networks, and defense regulation. With a particular focus on resistance to major fungal, bacterial, and viral pathogens, Figure 2 illustrates modern breeding technologies, including conventional methods, marker-assisted selection, and transgenic approaches, which together have facilitated the development of the next generation of resistant varieties that can meet global food security challenges. By bridging fundamental research with practical breeding applications, we highlight pathways to develop rice varieties that can meet rising global demand while reducing agriculture’s environmental footprint, a crucial step toward achieving both food security and sustainability goals.

## 2. Rice–Pathogen Interactions at the Genetic Level

Strategic incorporation of resistance genes into rice varieties has become an essential approach for sustainable disease management, environmental protection, and reduced reliance on agrochemicals. Cutting-edge genome editing technologies, particularly the CRISPR-Cas platform, now allow precise modification of the rice genome to enhance defense responses to evolving pathogen populations [33]. Contemporary research efforts have successfully identified multiple genetic components that confer broad-spectrum resistance, including major *R* genes [34], defense regulatory elements [35], and quantitatively inherited chromosomal regions (QTLs) [36]. Notably, comprehensive genome-wide association analyses have identified key QTL clusters on chromosomes 5, 6, and 9 associated with durable resistance to the bacterial wilt pathogen, providing valuable genetic targets for breeding programs [37].

Recent studies have highlighted the crucial role of microRNAs (miRNAs) in regulating rice immune responses against bacterial and fungal pathogens. For instance, Osa-miR398 has been shown to negatively regulate rice blast resistance by targeting genes involved in reactive oxygen species (ROS) detoxification, including CSD1 and CSD2, thus modulating the oxidative burst during *M. oryzae* infection [38]. Similarly, Osa-miR164a has been implicated in enhancing resistance to *Xanthomonas oryzae* pv. *oryzae* by targeting NAC transcription factors involved in programmed cell death and pathogen defense [39]. These miRNA-target modules demonstrate the layered complexity of post-transcriptional regulation in rice–pathogen interactions and provide promising molecular targets for genetic improvement strategies focused on durable resistance. Deployment of natural resistance genes in commercial rice varieties is often challenging, as they are associated with reduced yield performance and rice quality parameters. This requires a thorough characterization of two fundamentally different resistance mechanisms: qualitative resistance, mediated by a single major gene with a dominant effect, and quantitative resistance, involving the cumulative effects of multiple minor-acting genes. A proper understanding of these complementary systems is essential for breeding rice varieties that achieve the optimal synergy between robust disease resistance and superior agronomic performance.

### 2.1. Qualitative Resistance Mechanisms in Rice

Qualitative resistance in rice is marked by clear phenotypic differences that follow predictable Mendelian inheritance patterns and are usually controlled by a small number of genes with major effects. These significant *R* genes provide strong defense against specific pathogen strains and can be efficiently identified and mapped through genetic screens [40]. A well-known example is the Sarawak landrace rice cultivar, where researchers isolated a resistance gene effective against the rice blast fungus, confirming the qualitative nature of this resistance [41]. This type of resistance operates on the gene-for-gene model, where the interaction between the plant’s *R* genes and the pathogen’s avirulence (*Avr*) genes triggers a hypersensitive response that blocks pathogen infection [37]. However, pathogens can evolve to overcome this resistance, as seen with rice blast strains that have defeated resistance conferred by genes like *Pi2* and *Pi9* through mutations in their Avr genes [24,26].

The genetic basis of qualitative resistance involves two key classes of proteins encoded by *R* genes: receptor-like kinases (RLKs) and nucleotide-binding leucine-rich repeat (NLR) proteins, both crucial for plant immunity [42]. RLKs recognize general pathogen-associated molecular patterns (PAMPs), while NLRs detect specific pathogen effectors. These recognition events activate two defense pathways, PAMP-triggered immunity and effector-triggered immunity [43], leading to the production of reactive oxygen species and antimicrobial compounds that restrict pathogen growth [44]. NLR proteins are particularly effective at inducing localized cell death to contain infections, a key mechanism in preventing pathogen spread [45]. Research on the *OsSPK1-OsRac1-RAI1* signaling pathway has revealed a conserved defense mechanism among various NLR proteins in rice [46]. In the rice genome, genes encoding RLKs and NLRs are often clustered in disease resistance hotspots, which frequently overlap with quantitative trait loci (QTLs) linked to disease resistance [47]. For example, QTL-seq analysis identified key resistance regions on chromosomes 1, 9, and 10 against rice ear blight, with RLK and NLR genes as the primary candidates [48].

A major limitation of qualitative resistance is its race specificity, making it susceptible to evolving pathogens [49]. *Xanthomonas oryzae* pv. *oryzae* (*Xoo*), the bacterium causing bacterial blight, is a classic example, as it can adapt to overcome certain *R* gene defenses. Traditional farming has used multi-line breeding, growing different rice varieties with distinct resistance genes to reduce pathogen selection pressure. Modern breeding programs improve resistance durability by stacking multiple *R* genes into a single variety [50,51]. The Zhachanglong rice variety is an example, combining *Xa3/Xa26*, *Xa22*, and *Xa31* genes for broad-spectrum resistance against multiple Xoo strains [52]. Advances in genetic engineering have also been promising. The N46(*Xa23R*) rice line, developed in Brazil, contains an effector-binding element in the xa23 gene promoter, providing resistance against multiple *Xoo* and *Xoc* strains without affecting yield [53].

### 2.2. Quantitative Resistance in Rice: Key Genetic Advances

Quantitative resistance in rice is governed by a complex network of multiple genetic loci, each contributing small but cumulative effects to overall disease resistance [54]. Unlike qualitative resistance that depends on a single major gene for complete protection against specific pathogens, quantitative resistance offers broader and more durable protection that is less susceptible to pathogen adaptation [55]. This form of resistance involves numerous genes participating in pathogen recognition, signal transmission, and hormonal regulation within the plant [54]. The foundation of quantitative resistance lies in quantitative trait loci (QTLs), which help mitigate diseases like rice streak necrosis virus (RSNV) and false smut [56]. These QTLs are distributed across various chromosomal regions and influence different defensive mechanisms to reduce disease impact. Significant progress has been made in identifying and characterizing these QTLs, providing insights into the genetic architecture of disease resistance. For instance, the qHBV4.1 locus has been established as a major contributor to resistance against white heads disease [57]. Research on false smut has also uncovered genomic regions rich in resistance genes, with the QTL qRFSr9.1 on chromosome 9 showing particularly strong phenotypic effects, making it a prime target for breeding programs. These QTLs correlate with critical resistance indicators such as infection rates per plant and smut ball formation per panicle [58].

Additional studies have deepened our understanding of resistance mechanisms. Research by Inoue and Hayashi demonstrated that the qPbm11 QTL, which provides blast resistance in Miyazaki Mochi varieties, functions independently of the known Pb1 gene [59]. This finding suggests that combining multiple QTLs through gene pyramiding could enhance blast resistance. Similarly, genome-wide association studies by Zhang et al. [60] highlighted the significance of jasmonic acid and salicylic acid pathways in regulating resistance to sheath blight, suggesting that these hormonal pathways may be potential targets for breeding strategies.

Further discoveries include the identification of qRFS12.01, a novel QTL associated with false smut resistance, emphasizing the value of quantitative resistance given the absence of completely resistant rice varieties [61,62]. Through QTL analysis, researchers mapped a new resistance gene, OsDRq12, to chromosome 12. This gene belongs to the NLR family and significantly boosts disease resistance in rice cultivars [63]. Large-scale genome-wide association studies have identified 74 QTLs linked to resistance against panicle blight and leaf blight, with the qPBR1 locus showing particularly strong, development-stage-independent resistance [64]. Research by Okello et al. [37], using the MAGIC indica panel, pinpointed three QTLs on chromosomes 5, 6, and 9 that confer broad resistance against African bacterial blight strains, underscoring the need for novel resistance genes against evolving pathogens. An important development has been the strategic combination of multiple QTLs, particularly those conferring resistance to major diseases like blast, sheath blight, and bacterial blight, into clusters within specific chromosomal regions (Table 2) [43,65]. This clustering not only refines the genetic targeting of QTLs but also facilitates the identification of candidate genes for breeding programs. These advances enable scientists to substantially enhance rice resistance and develop more sustainable disease management approaches in rice cultivation [66,67].

## 3. Gene-for-Gene Concept in Rice Disease Resistance

The gene-for-gene concept forms a fundamental framework for understanding rice–pathogen interactions and plays a crucial role in developing disease-resistant rice varieties. Originally proposed by Harold Flor in the 1950s [75], this model establishes that specific *R* genes in the host plant interact with corresponding *Avr* genes in the pathogen. Extensive research in rice has validated this principle through studies of its interactions with major pathogens, including *Xoo* and *M. oryzae*, revealing the intricate molecular interplay between host defenses and pathogen virulence mechanisms [76].

**Figure 3:**
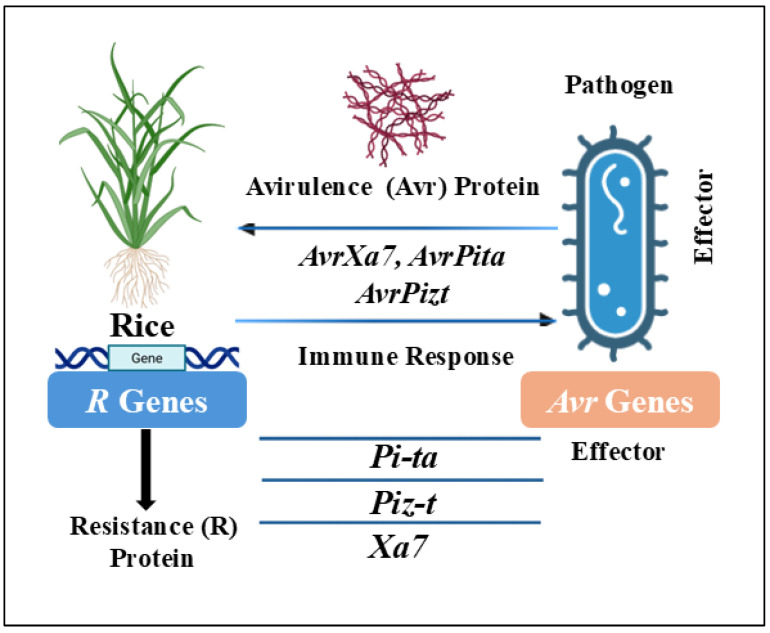
Schematic representation of effector-triggered immunity (ETI) in rice, illustrating the interaction between pathogen-derived avirulence (Avr) proteins and host resistance (R) proteins. Recognition of effectors such as *AvrXa7*, *AvrPita*, and *AvrPiz-t* by corresponding R proteins (*Xa7*, *Pi-ta*, and *Piz-t*, respectively) activates a robust immune response in the host plant. These gene-for-gene interactions are well characterized in *Xanthomonas oryzae* pv. *oryzae* and *Magnaporthe oryzae* systems [77,78].

This concept has been particularly well documented in rice’s defense against bacterial blight and blast disease, where the recognition of pathogen Avr proteins by plant R proteins triggers a strong immune response (Figure 3). The interaction follows a precise molecular recognition system, where the presence of both matching *R* and *Avr* genes leads to resistance, while the absence or mutation in either component can result in susceptibility. These findings have not only confirmed Flor’s original hypothesis but have also provided critical insights for breeding programs aiming to develop durable resistance in rice cultivars through the strategic deployment of *R* genes. The elucidation of genetic mechanisms such as gene-for-gene interactions and the identification of specific resistance loci (e.g., *Pi* and *Xa* genes) have provided a strong molecular foundation for modern rice breeding. These insights not only clarify how plants mount defense responses but also guide the strategic use of breeding technologies, such as marker-assisted selection and CRISPR-based genome editing to introduce, pyramid, or fine-tune resistance traits in elite cultivars. The following sections build upon these genetic principles by examining how breeders translate them into practical strategies to develop resilient, high-yielding rice varieties capable of withstanding evolving pathogen threats.

### 3.1. Gene-for-Gene Resistance Mechanisms in Rice Against Xanthomonas oryzae

The interaction between rice and *Xanthomonas oryzae* (*Xoo*) operates through a precise gene-for-gene relationship, where specific *R* genes in rice recognize corresponding *Avr* genes in the pathogen [79]. This molecular recognition system serves as a cornerstone of rice immunity against bacterial blight. A well-characterized example is the *Xa23* gene in rice, which confers resistance to *Xoo* strains carrying the matching *avrXa23* gene [53]. Similarly, other *R* genes, including *Xa3*, *Xa2*, *xa5*, and *xa8*, recognize their respective *Avr* counterparts (*avrXa3*, *avrXa2*, *avrxa5*, *avrxa8*) and provide resistance in compatible rice varieties [24]. When an R protein detects its cognate Avr effector, it triggers a robust immune response. For instance, *Xa3* detects pathogen-associated molecular patterns (PAMPs) on bacterial membranes, activating localized defense reactions that restrict pathogen spread [80]. This often leads to hypersensitive cell death at infection sites, creating a physical barrier against further invasion.

Central to this process is *Xoo*’s type III secretion system (T3SS), which delivers Avr effectors directly into rice cells [81]. Recent studies have elucidated key aspects of these interactions. For example, the Avr effector *Xa7* binds to the promoter region of the rice *Xa7* resistance gene, inducing a hypersensitive response that suppresses bacterial growth [82]. Similarly, research by Zou et al. [83] demonstrated how Avr recognition activates rice defense pathways, effectively halting disease progression. These insights highlight the potential for leveraging *R-Avr* interactions to engineer broad-spectrum resistance in rice breeding programs. A critical feature of rice–*Xoo* interactions involves transcription activator-like effectors (TALEs), which *Xoo* secretes via T3SS to manipulate host gene expression [84]. TALEs function as virulence factors by activating susceptibility genes or suppressing plant immunity [85]. However, rice has evolved countermeasure *R* genes like *Xa1*, *Xa10*, and *Xa23* that detect specific TALEs and mount a hypersensitive response to block infection [53].

This defense is sometimes circumvented by *Xoo* strains producing interfering TALEs (iTALEs), which disrupt *R* gene recognition and enable immune evasion [84]. Such adaptations underscore the ongoing evolutionary arms race between rice and *Xoo* [86]. The *avrBs3*/*pthA* gene family in *Xoo* plays a particularly significant role in modulating resistance. These genes, which may exist singly or in clusters within the pathogen genome, influence resistance patterns in rice [87]. The gene-for-gene model explains why specific cultivars are resistant to bacterial blight while others remain susceptible. Notably, some *R* genes (e.g., *Xa3* and *Xa21*) share signaling pathways, suggesting partially overlapping yet distinct defense mechanisms [88]. Many *R* genes, including *Xa3*, *Xa26*, and *Xa4*, encode receptor-like kinases (RLKs) that recognize PAMPs and initiate immune responses such as cell wall reinforcement and defense pathway activation [51]. These RLKs are pivotal components of rice immunity, and deciphering their interactions could inform strategies for developing disease-resistant rice varieties with durable immunity. By harnessing this knowledge, breeders can design rice cultivars with stacked *R* genes or edited promoter regions to outpace pathogen evolution and sustain crop protection.

### 3.2. Gene-for-Gene Resistance Mechanisms in Rice Against Magnaporthe oryzae

**Figure 4:**
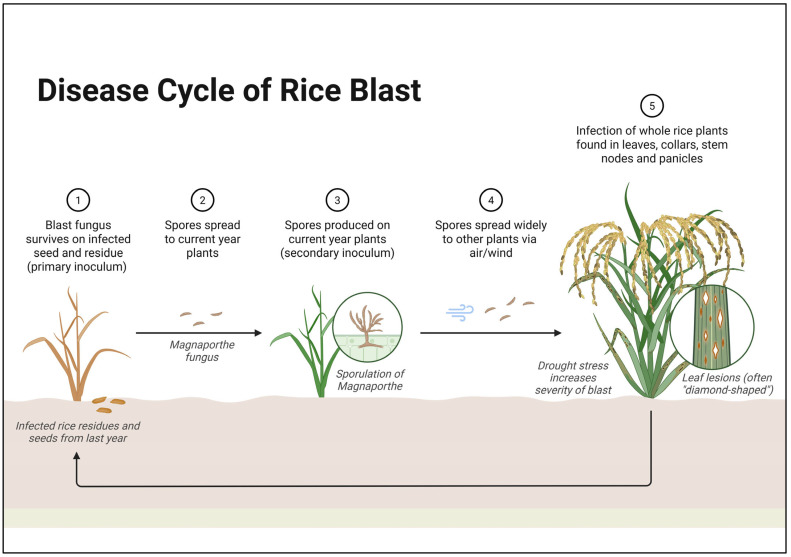
Life cycle of *M. oryzae*, showing key stages including spore germination, hyphal growth, lesion development, sporulation, and sexual reproduction via perithecium formation [90,91].

The genetic interactions between rice and the rice blast fungus *Magnaporthe oryzae* (formerly *M. grisea*) exemplify a sophisticated coevolutionary arms race. The rice blast fungus spreads through spores carried by the wind, germinating on rice seedlings and forming adhesive organs to penetrate the tissue. Inside the host, it causes damage, produces new spores, and completes this cycle in 5 to 7 days. The pathogen can survive in infected residues and seeds during the season, leading to recurrent outbreaks (Figure 4). At the core of this battle are specific *R* genes in rice, particularly the *Pi* genes (*Pi-ta*, *Pia*, *Pii*) that recognize the corresponding *Avr* genes in the pathogen [5]. When a rice plant carrying a *Pi* gene encounters a blast strain with the matching Avr effector, it triggers a hypersensitive response that halts fungal invasion. The *Pi-ta*/*AVR-Pita* interaction serves as a paradigm: the cytoplasmic NLR protein encoded by *Pi-ta* directly binds the *AVR-Pita* effector, initiating defense responses, including localized cell death, to contain the infection [89].

This recognition system drives continuous adaptation on both sides. Pathogen populations evolve through *Avr* gene mutations and haplotype diversification to evade detection, as seen in variants like *AvrPi54* and *AvrPii* [92]. The emergence of novel effectors (e.g., *AVR-Pi9*, *AVR-Mgk1*) demonstrates the pathogen’s ability to circumvent existing resistance, necessitating ongoing surveillance and adaptive breeding. To date, researchers have documented over 30 rice *R* genes and 12 *M. oryzae Avr* genes, revealing diverse recognition mechanisms [44]. While some NLR receptors like *Pi-ta* detect effectors through direct binding, others (e.g., *Pik*) employ integrated decoy domains for indirect recognition, as shown in the *Pik*/*AVR-Pik* and *Pia*/*AVR-Pia* systems [93,94]. The evolutionary dynamics vary across rice subspecies, with indica and japonica cultivars often exhibiting distinct resistance spectra due to differential pathogen adaptation. Breeding strategies now emphasize pyramiding multiple *Pi* genes (e.g., *Pi2*, *Pi9*, *Pi54*) to create durable, broad-spectrum resistance [95]. The application of *Pi* genes in breeding programs has led to the development of several successful rice cultivars with enhanced resistance to blast. For instance, the cultivar IRBL9-W incorporates the *Pi9* gene and has shown durable resistance to a wide range of *M. oryzae* strains. Similarly, Putta Basmati 1509, which combines *Pi2* and *Pi54*, has been widely adopted in India due to its broad-spectrum blast resistance. These examples demonstrate how knowledge of specific *R-Avr* interactions can be harnessed to develop and deploy resistant varieties in real-world agriculture. This approach leverages the observation that combined *R* genes can collectively block diverse fungal strains. The continued identification of novel *Avr* genes and their interactions with host NLR proteins remains critical for developing next-generation blast-resistant rice, particularly as climate change accelerates pathogen evolution. These efforts are further supported by advances in effectoromics, which enable systematic screening of *Avr* gene diversity in field populations to predict and counteract emerging virulence trends [96].

## 4. MAPK Signaling in Rice Immunity: Key Roles in Defense Against *Xoo*

Mitogen-activated protein kinase (MAPK) cascades serve as central regulators of rice immune responses against *Xoo* infection. These signaling pathways are rapidly activated upon pathogen recognition, initiating phosphorylation cascades that amplify defense mechanisms [97]. Transcriptomic analyses reveal that MAPK-mediated signaling drives critical defensive processes, including cell wall fortification and biosynthesis of antimicrobial compounds [98]. Key components like OsMKK6 and OsMPK4 form an interconnected network that enhances resistance to bacterial blight [97]. Within hours of *Xoo* infection, MAPKs such as OsMPK3, OsMPK4, and OsMPK6 are activated, implicating their role in early defense responses [99]. These kinases phosphorylate transcription factors, including WRKY13 and WRKY45, which subsequently orchestrate the expression of defense-related genes [100]. This coordinated action bridges local and systemic immunity, enabling comprehensive pathogen resistance [101,102].

Concurrently, MAPK signaling induces structural defenses, such as callose deposition and lignin biosynthesis, thereby reinforcing physical barriers against bacterial invasion [91]. However, *Xoo* employs counterstrategies to subvert these defenses, beyond its well-characterized TAL effectors; the pathogen secretes additional virulence factors that actively suppress MAPK activation [103]. This highlights the dynamic interplay between rice immune signaling and bacterial evasion tactics. Deciphering the architecture of the MAPK cascade and its manipulation by *Xoo* provides critical insights for developing novel resistance strategies [104]. By targeting specific nodes within this pathway, either through genetic engineering or precision breeding, researchers can potentially engineer rice varieties with enhanced, durable resistance to bacterial blight. These approaches could focus on stabilizing MAPK activation or blocking effector-mediated suppression to maintain robust immune responses.

## 5. Conventional Breeding for Disease-Resistant Rice: Challenges and Advances

Conventional breeding has long served as the foundation for developing disease-resistant rice varieties, helping to safeguard yields and ensure global food security. Through methods such as phenotypic selection, controlled crossing, and backcrossing, breeders have successfully introduced resistance to major diseases like rice blast, bacterial blight, and sheath blight [105,106]. A key strategy involves transferring resistance genes from wild relatives or naturally resistant landraces into high-yielding but susceptible elite cultivars. Notable examples include the introgression of the *Pi2* and *Pi9* blast resistance genes into commercial rice varieties, significantly enhancing protection against this devastating fungal pathogen [107]. Despite its successes, conventional breeding faces several limitations. A major challenge is linkage drag, where undesirable traits from donor plants are inadvertently transferred alongside resistance genes. This can negatively impact critical agronomic qualities such as yield potential, grain quality, or stress tolerance, reducing farmer adoption of new varieties [108]. Additionally, traditional breeding is inherently slow, often requiring 8 to 12 generations of meticulous crossing and backcrossing to achieve the ideal combination of disease resistance and superior agronomic performance [109]. Another critical issue is the durability of resistance: pathogens can rapidly evolve to overcome single-gene resistance introduced through conventional methods, leading to breakdowns in field efficacy [110]. Furthermore, balancing resistance with essential traits like high productivity remains an ongoing challenge, as some resistance mechanisms may incur fitness costs or alter plant physiology in ways that compromise yield [111].

To address these constraints, modern breeding has adopted marker-assisted selection as a complementary tool. By using DNA markers linked to resistance genes, breeders can precisely track and select desired traits while minimizing linkage drag [112]. This approach accelerates the development of resilient varieties that maintain yield and quality, bridging the gap between traditional breeding and advanced biotechnological solutions. While conventional methods remain indispensable, integrating MAS and other precision breeding techniques offers a pathway to more efficient and sustainable disease management in rice cultivation.

### 5.1. Marker-Assisted Selection in Rice Breeding: Successes, Challenges, and Future Directions

Marker-assisted selection (MAS) has revolutionized rice breeding by enabling precise introgression of resistance genes into elite varieties, significantly enhancing their ability to combat major pathogens [113]. This approach has proven particularly effective against devastating diseases like rice blast and bacterial blight, allowing breeders to develop cultivars with durable, broad-spectrum resistance [28]. The technique’s success is evident in several landmark achievements: in China, resistant lines such as Huahui 7713 and Huahui 3006 were developed by incorporating the *Pigm*, *Bph6*, and *Bph9* genes, leading to high-yielding hybrids like Weiliangyou 7713 that maintain both disease resistance and superior grain quality [28]. Similar success was seen in India, where MAS introduced *Xa21*, *xa13*, and *xa5* into aromatic rice varieties, creating lines with robust bacterial blight resistance without compromising desirable traits [15].

However, MAS faces significant challenges that limit its effectiveness. The rapid evolution of pathogens can render resistance genes ineffective over time, as seen with some Xanthomonas oryzae strains that have overcome Xa23-mediated resistance [53]. Additionally, the process of stacking multiple resistance genes remains technically demanding and time-consuming, complicated by genetic interactions and environmental influences [114]. Perhaps most critically, MAS typically targets specific pathogens or strains, leaving crops susceptible to emerging diseases or new pathogen variants [115]. These limitations highlight the need for complementary approaches to ensure durable resistance. Looking ahead, the integration of MAS with emerging technologies offers promising solutions [116]. CRISPR/Cas9 genome editing enables precise modification of resistance genes or their regulatory elements, potentially broadening and stabilizing resistance [117]. High-throughput phenotyping accelerates the identification and validation of resistance traits, while combining MAS with integrated pest management strategies could provide more sustainable disease control [118]. Despite its challenges, MAS remains an indispensable tool in rice breeding, though its long-term success will depend on strategic integration with these advanced approaches and careful consideration of region-specific agricultural challenges. While MAS has significantly enhanced breeding precision, several limitations persist. Stacking multiple resistance genes remains technically complex due to epistatic interactions and linkage drag, where undesirable traits may co-segregate with beneficial alleles. Environmental interactions may also affect the expression of QTLs or resistance genes, leading to genotype-by-environment variability in disease response. To overcome these hurdles, breeders are now integrating MAS with genomic selection (GS) and high-throughput phenotyping platforms, which allow for the simultaneous selection of multiple traits with greater predictive power. Furthermore, the use of tightly linked or gene-specific markers, such as SNPs derived from resistance gene sequences, has improved selection accuracy and reduced linkage drag. These advances make MAS more robust, particularly in combination with other precision breeding tools.

### 5.2. CRISPR/Cas9: A Revolutionary Tool for Enhancing Disease Resistance in Rice

**Figure 5:**
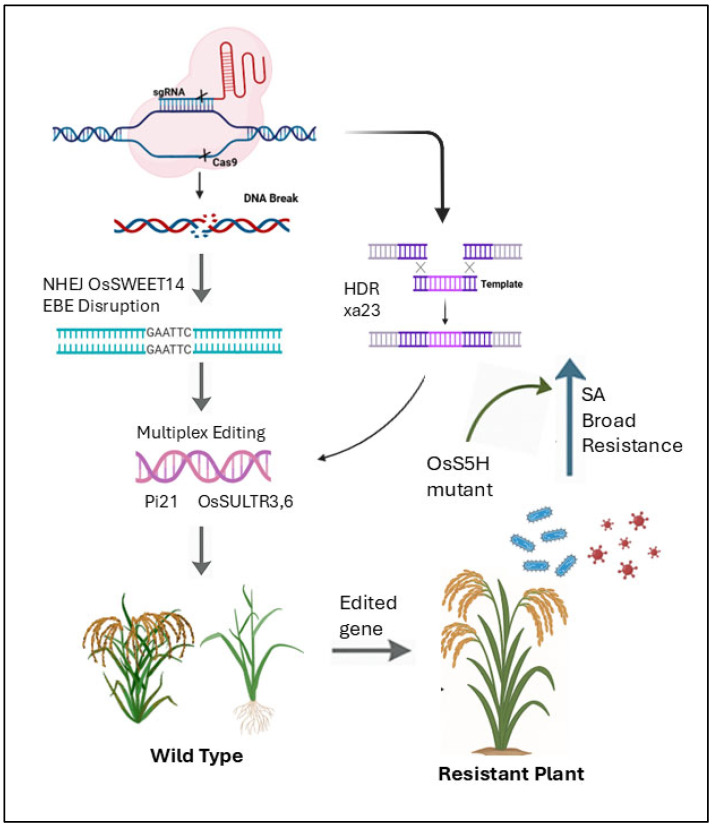
Genome editing strategies for developing disease-resistant rice. The figure illustrates key approaches, including (1) disruption of susceptibility genes (e.g., *NHEJ*, *OsSWEET14*) [32], (2) homology-directed repair (HDR) for introducing resistance alleles (e.g., *xa23*) [126], (3) multiplex editing of multiple targets (e.g., *Pi21*, *OsSULTR3,6*), and (4) generation of broad-spectrum resistance through edited genes (e.g., *OsS5H mutant*), compared to wild-type plants. Edited lines show enhanced resistance to pathogens [127].

CRISPR/Cas9 technology has revolutionized rice breeding by enabling precise genome editing to enhance disease resistance, particularly against bacterial blight [53]. The system works by using a designed single-guide RNA (sgRNA) to direct the Cas9 nuclease to specific DNA sequences, creating double-strand breaks that are subsequently repaired through either error-prone non-homologous end joining (NHEJ) or precise homology-directed repair (HDR) (Figure 5) [119]. This approach has successfully generated rice plants with improved resistance to both bacterial blight and rice blast diseases [120]. A groundbreaking application involves editing the *OsSWEET14* susceptibility gene, which *Xoo* exploits through its transcription activator-like effectors (TALEs) [121]. Researchers used CRISPR/Cas9 to disrupt effector-binding elements (EBEs) in the *OsSWEET14* promoter of Super Basmati rice, creating edited lines (SB-E1 to SB-E4) that showed significantly reduced lesion lengths and enhanced resistance compared to wild-type plants [122]. This strategy demonstrates how targeted editing of host susceptibility factors can confer resistance without introducing foreign DNA, offering a sustainable solution for disease management [123]. In fungal disease control, deletion of the *Bsr-d1* susceptibility gene enhanced blast resistance in Japonica rice, with protective effects evident from the seedling stage. Multiplex editing has proven particularly powerful, as shown by simultaneous modification of *Pi21* and *OsSULTR3;6* genes, which conferred dual resistance to blast and bacterial leaf spot while preserving yield potential [124]. Researchers have also successfully targeted systemic defense pathways, such as creating *OsS5H* mutants that exhibit broad-spectrum resistance through salicylic acid-mediated defense activation [125].

CRISPR/Cas9’s precision allows for sophisticated modifications like promoter engineering, exemplified by editing the xa23 gene promoter to incorporate multiple EBEs, resulting in durable resistance to bacterial blight and streak [53]. Importantly, these genetic improvements can be achieved without compromising plant growth or grain quality. The integration of CRISPR technology with conventional breeding and other biotechnological tools presents a comprehensive strategy for developing next-generation rice varieties that combine high productivity with robust, durable disease resistance and a critical advancement for global food security in an era of evolving pathogen threats. Despite the promise of CRISPR/Cas9 in rice disease resistance breeding, the technique is not without limitations. Off-target mutations, unintended edits in non-target regions, pose a risk of unwanted phenotypic changes or compromised plant fitness. Breeders are actively mitigating these concerns by employing high-fidelity Cas9 variants (e.g., SpCas9-HF1, eSpCas9) and guide RNA design tools that increase target specificity. Additionally, delivery methods such as ribonucleoprotein complexes (RNPs) reduce the risk of stable integration and transiently expose the genome to editing components, further minimizing off-target effects. Beyond technical issues, regulatory hurdles, especially in regions where genome-edited crops are treated similarly to GMOs, remain a significant challenge. To address this, researchers are focusing on non-transgenic genome editing approaches, such as using CRISPR to generate edits without integrating foreign DNA, which may ease regulatory acceptance in some jurisdictions.

## 6. Development and Impact of Disease-Resistant Rice Varieties

Disease-resistant rice varieties represent a strategic breakthrough in combating major rice pathogens through the targeted incorporation of resistance genes that strengthen the plant’s innate defense mechanisms. These genetically enhanced cultivars have substantially decreased dependence on chemical pesticides while promoting sustainable crop production and boosting yield stability [128]. By integrating specific resistance traits, these varieties maintain consistent productivity even under significant disease pressure, establishing themselves as indispensable components of modern rice cultivation systems.

Several high-performing varieties exemplify this approach through their effective management of devastating diseases like bacterial blight and rice blast [129]. These cultivars, often developed through meticulous breeding programs, showcase how genetic resistance can be practically deployed to prevent disease outbreaks and minimize yield losses. Their success stems from incorporating well-characterized resistance genes that trigger robust immune responses upon pathogen recognition. The effectiveness of these varieties is evident in their widespread adoption across different rice-growing regions, where they have demonstrated reliable performance against evolving pathogen populations. Table 3 summarizes key disease-resistant rice varieties along with their incorporated resistance genes, highlighting their specific roles in controlling major rice diseases. These examples underscore the critical need for ongoing research and breeding innovation to develop new resistant varieties capable of countering emerging pathogen strains while maintaining optimal agronomic performance. The continued development and deployment of such varieties remain essential for ensuring global rice security in the face of persistent and evolving disease threats.

## 7. Environmental Impact of Disease-Resistant Rice Varieties

The development of disease-resistant rice varieties represents a critical advancement in sustainable agriculture, offering a powerful solution to reduce pesticide dependence while addressing major threats to global rice production like bacterial leaf blight and rice blast [104]. These genetically enhanced cultivars provide multiple environmental benefits, primarily through dramatically decreased pesticide application. This reduction lowers farming costs while preventing chemical runoff that contaminates waterways and soils, thereby protecting aquatic ecosystems and maintaining soil health [28,137]. Beyond pollution control, disease-resistant varieties actively promote biodiversity conservation in rice-growing regions. By minimizing broad-spectrum pesticide use, they safeguard beneficial insects, soil microbes, and aquatic organisms that form the foundation of healthy agroecosystems [138]. This preserved biodiversity enhances natural pest control, improves soil fertility, and increases ecosystem resilience to climate variability, all crucial factors for sustainable rice production [139,140].

However, these benefits must be balanced against potential ecological risks, particularly concerning gene flow to wild rice populations. Uncontrolled transfer of resistance genes through cross-pollination could alter the genetic diversity of wild relatives, potentially compromising their natural adaptive capacity to environmental stresses [141]. Such genetic contamination might disrupt ecological balances and reduce the genetic reservoirs needed for future crop improvement [142]. To maximize benefits while minimizing risks, strategic implementation is essential. This includes maintaining buffer zones around resistant varieties, continuous monitoring of wild populations, and developing containment strategies for engineered genes. When properly managed, disease-resistant rice varieties serve as a cornerstone of sustainable intensification, simultaneously boosting food security and environmental protection [143]. Their responsible deployment demonstrates how agricultural innovation can align with ecological preservation to meet the dual challenges of productivity and sustainability in rice farming systems.

## 8. The Future of Disease-Resistant Rice: Challenges and Opportunities

As global rice demand rises, developing disease-resistant varieties is essential to safeguarding food security. The field faces both transformative opportunities and complex challenges that demand innovation and cross-disciplinary collaboration. Cutting-edge gene editing tools like CRISPR/Cas9 have revolutionized rice breeding, enabling precise modifications to disrupt susceptibility genes or introduce robust resistance traits. Yet, hurdles remain; improving editing efficiency, minimizing off-target effects, and navigating regulatory landscapes must be addressed to fully realize this technology’s potential. Climate change adds urgency to these efforts, as shifting temperatures and weather patterns alter pathogen dynamics and geographic ranges. Future rice varieties must combine disease resistance with resilience to abiotic stresses like drought, salinity, and extreme heat. This requires integrated breeding strategies that simultaneously target biotic and abiotic pressures, ensuring adaptability in a changing environment. Pathogen evolution remains a persistent threat, necessitating durable solutions. Approaches like gene pyramiding (stacking multiple *R* genes), harnessing QTLs for stable partial resistance, and mining wild rice germplasm for novel resistance sources will be critical. These strategies can extend the longevity of resistance traits while reducing reliance on chemical controls. Breakthroughs in multiomics technologies (genomics, transcriptomics, proteomics, and metabolomics) promise to deepen our understanding of rice–pathogen interactions. By uncovering new resistance mechanisms and precise molecular targets, these tools can accelerate the development of precision-bred varieties with enhanced defenses.

However, technological advances must align with sustainable agricultural practices. Reducing pesticide dependence through resistant varieties should complement integrated pest management (IPM) systems, ensuring ecological balance. Equally important is addressing societal and regulatory concerns, fostering transparency, engaging stakeholders, and establishing science-based policies will be key to deploying these innovations globally. The path forward hinges on balancing innovation with responsibility. By uniting advanced breeding tools, climate-smart strategies, and ecological stewardship, next-generation rice varieties can deliver both high yields and long-term sustainability, securing food systems for future generations.

## 9. Conclusions

The field of disease-resistant rice development has undergone transformative progress through genetic and molecular breakthroughs. The gene-for-gene model has served as a cornerstone for deciphering plant–pathogen interactions, guiding the identification and utilization of critical resistance genes like the *Xa* series against bacterial blight and *Pi* genes against rice blast. These discoveries have revolutionized breeding methodologies, enabling precision strategies such as marker-assisted selection and gene pyramiding to create robust, high-performing rice varieties. Emerging technologies, particularly CRISPR-based genome editing, have further expanded the toolkit for enhancing disease resistance. By enabling targeted modifications of susceptibility genes or regulatory elements, these approaches allow for the development of resistant cultivars without compromising yield or quality. Coupled with growing insights into plant immune mechanisms, from pathogen recognition to defense signaling cascades, these innovations are making resistance breeding more efficient and effective. However, the rapid evolution of pathogens threatens to overcome single-gene resistance, while the polygenic nature of quantitative resistance complicates breeding efforts. Additionally, integrating disease resistance with other vital traits, such as drought tolerance or grain quality, remains a delicate balancing act. Climate change exacerbates these challenges by altering pathogen distributions and infection dynamics, demanding more adaptable varieties. Moving forward, a multi-disciplinary, integrated approach will be essential. Combining traditional breeding with cutting-edge molecular tools, genomic selection, and high-throughput phenotyping can accelerate the development of durable, broad-spectrum resistance. Equally critical is the adoption of sustainable agricultural practices, such as diversified cropping systems and integrated pest management, to prolong resistance efficacy. Success will hinge on strengthened collaboration among breeders, pathologists, molecular biologists, and agronomists to address these complex, interconnected challenges. By leveraging advances in science while maintaining ecological and agronomic balance, the global community can ensure rice remains a resilient, productive staple crop in the face of evolving threats to food security.

## Figures and Tables

**Table 1:** Common rice diseases: etiology, symptomatology, and financial impact.

Disease	Pathogen	Symptoms	Region and Year	Economic Impact	References
Rice Blast	*Magnaporthe oryzae*	Leaf lesions, neck rot, panicle blast	Mid-South USA, 2016	Annual producer gains of USD 69.34 million with blast-resistant rice adoption.	[10]
Bacterial Blight	*Xanthomonas oryzae* pv. *oryzae*	Water-soaked lesions, wilting, yellowing of leaves	India, 1980s	Yield losses up to 30% in the Punjab region.	[11]
Sheath Blight	*Rhizoctonia solani*	Lesions on leaf sheaths, lodging, reduced grain quality	India (Uttar Pradesh), 2015	Yield losses ranged between 14.3% and 39.7% across surveyed districts.	[12]

**Table 2:** Major QTLs and genes associated with resistance to important fungal and bacterial pathogens in rice.

Gene/QTLs	Pathogen	Role	References
*qSB-9*	*R. solani*	Decreases the severity of sheath blight infection	[68]
*qSBR11*	*R. solani*	Promotes sheath blight resistance	[69]
*hb9-2*	*R. solani*	Imparts partial resistance to sheath blight	[70]
*qBlsr5a*	*Xoo*	Increases host resistance to bacterial leaf streak	[71]
*qSBR11-1*	*Xoo*	Provides durable resistance across bacterial blight races	[72]
*Pi21*	*M. oryzae*	Offers partial resistance to *M. oryzae*	[73]
*Pi35*	*M. oryzae*	Provides partial resistance to *M. oryzae*	[74]

**Table 3:** Prominent disease-resistant rice cultivars and their role in controlling plant diseases.

Resistant Genes	Variety	Disease	References
*Pi-1*, *Pi-2*, *Pi-33*	C101A51	Rice blast	[130]
*Pi-2*, *Pi-54*	Puta Basmati 1509	Rice blast	[131]
*Pi9*	IRBL9-W	Rice blast	[30]
*Xa23*, *Pi9*	Super 1000	Bacterial blight, rice blast	[132]
*Xa21*	IR72	Bacterial blight	[133]
*Xa21*, *Xa23*	Minghui 63	Bacterial blight	[134]
*X4*, *X5*, *X13*, *X21*	IR36	Bacterial blight	[89]
*Xa21*, *xa13*, *Xa5*	Samba Mahsuri	Bacterial blight	[135]
*Xa21*	IRBB21	Bacterial blight	[136]

## Data Availability

Not applicable.
